# Processing of Hand-Related Verbs Specifically Affects the Planning and Execution of Arm Reaching Movements

**DOI:** 10.1371/journal.pone.0035403

**Published:** 2012-04-20

**Authors:** Giovanni Mirabella, Sara Iaconelli, Silvia Spadacenta, Paolo Federico, Vittorio Gallese

**Affiliations:** 1 Department of Neuroscience, Istituto Neurologico Mediterraneo Neuromed, Pozzilli (IS), Italy; 2 Department of Experimental Medicine, University of L'Aquila, L'Aquila, Italy; 3 Department of Physiology and Pharmacology, University of Rome La SapienzaRome, Italy; 4 Department of Neuroscience, Section of Physiology, University of Parma, Parma, Italy; 5 IIT (Italian Institute of Technology) Brain Center for Social and Motor Cognition, Parma, Italy; French National Centre for Scientific Research, France

## Abstract

Even though a growing body of research has shown that the processing of action language affects the planning and execution of motor acts, several aspects of this interaction are still hotly debated. The directionality (i.e. does understanding action-related language induce a facilitation or an interference with the corresponding action?), the time course, and the nature of the interaction (i.e. under what conditions does the phenomenon occur?) are largely unclear. To further explore this topic we exploited a go/no-go paradigm in which healthy participants were required to perform arm reaching movements toward a target when verbs expressing either hand or foot actions were shown, and to refrain from moving when abstract verbs were presented. We found that reaction times (RT) and percentages of errors increased when the verb involved the same effector used to give the response. This interference occurred very early, when the interval between verb presentation and the delivery of the go signal was 50 ms, and could be elicited until this delay was about 600 ms. In addition, RTs were faster when subjects used the right arm than when they used the left arm, suggesting that action–verb understanding is left-lateralized. Furthermore, when the color of the printed verb and not its meaning was the cue for movement execution the differences between RTs and error percentages between verb categories disappeared, unequivocally indicating that the phenomenon occurs only when the semantic content of a verb has to be retrieved. These results are compatible with the theory of embodied language, which hypothesizes that comprehending verbal descriptions of actions relies on an internal simulation of the sensory–motor experience of the action, and provide a new and detailed view of the interplay between action language and motor acts.

## Introduction

According to the theory of embodied language [1]–[4], language comprehension relies on an internal enactment of the sensory–motor experience associated with the presented word or sentence; as such, this process would involve the same neural systems used when perceiving or acting [2], [3]. Thus the understanding of action-related words, such as *to grasp*, would require the activation of the motor schema underlying the execution of the same act. Evidence in support of this claim has come from several studies. First of all, functional brain imaging (fMRI) studies have shown that either single verbs (e.g. [5]) or sentences [6], [7] describing concrete actions performed with the mouth, the hand or the leg elicited the activation of clusters along the premotor cortex in an effector-specific manner. Furthermore, Boulenger et al. [8] have shown that a somatotopic activation of the premotor cortex can be observed not only for concrete sentences but also for abstract sentences including action words.

Using transcranial magnetic stimulation, Buccino et al. [9] showed that, after the stimulation of the hand representation of the left motor cortex, motor-evoked potentials (MEPs) have a reduced amplitude when recorded from right-hand muscles while the subject is listening to sentences expressing hand-related actions. Likewise, MEPs recorded from right foot and/or leg muscles are modulated when listening to sentences expressing foot-related actions.

Behavioral studies have also provided evidence that language processing interacts with actions. Glenberg & Kaschak [10] showed that comprehending a sentence describing an action in a given direction (e.g. “Close the drawer”, which implies an action away from the body) facilitated a movement in the same direction and slowed down a movement in the opposite direction. This phenomenon has been named action-sentence compatibility effect (ACE). A different type of interaction between action and language was found using sentences which did not imply any direction of movement. For instance Buccino et al [9], exploiting a go/no-go paradigm in which healthy participants were required to press a button when they heard a sentence expressing either hand or foot actions and to refrain from moving when an abstract verb was presented, found that responses were slower when the effector used for responding was the same as that involved in the action expressed by the sentence. Thus they suggested the existence of a somatotopy during verb processing. Similar results were obtained by Sato et al. [11] exploiting an analogues go/no-go paradigm but employing single action verbs. Again, they found that RTs increased whenever the action expressed by the verb involved the same effector used to give the motor response (interference effect). Finally, Boulenger et al [12] found that subliminal presentation of action words did not change the RTs of reaching movements but did alter their kinematic parameters.

Overall, the available data suggest that listening to or reading language material referring to actions modulates both motor responses and the activity of cortical motor areas. Together these findings can be interpreted as evidence of the involvement of the cortical motor system in action-language understanding (for a different view see [13]).

However, there are several gaps in the understanding of the link between action-language processing and motor responses. For instance, it is unclear whether processing motor-related language induces facilitation or interference when the action described has to be performed. This relationship seems to change according to the type of stimuli administered and to the type of task employed. Whenever comprehension of a sentence which implies directionality is required an ACE is obtained. When participants are required to make a semantic judgment either of a sentence [9] or of a single verb [11] an interference effect occurs. Lexical decision tasks exploiting single verbs have provided quite varying results. Willems et al. [14] did not find differences in terms of correct responses between manual and non-manual action verbs in right- and left-handers. Neininger & Pulvermuller [15] showed that significant differences in action-verb processing emerged only in patients with lesions of the right frontal lobe. Finally, Sato et al. [11] showed that when participants are required to carry out a lexical task the interference effect disappears.

To investigate the relationship between action execution and the processing of motor language material, we carried out four experiments using a modified version of the go/no-go paradigm and stimuli as described in Sato et al. [11] in order to extend their results to a number of unanswered issues. Firstly, we wanted to check whether the interference effect could be replicated asking participants to respond with reaching movements instead of simple key presses. Reaching movements have a higher ecological relevance in primates than key-presses because, outside neurophysiology laboratories, they allow physical interactions with the environment, thus leading to material outcomes such as those relating to food or tools. As a consequence, reaches are likely to require different neural processing from other relatively simpler movements. Furthermore reaching movements give the possibility to establish whether the interference effects affects a kinematic parameter such as the movement time (MT).

Secondly, along with the same line of reasoning we extended our analysis to the percentage of mistakes, with the idea that the motor–linguistic competition should also affect this aspect of the participants' performance.

Thirdly we wanted to make sure that the interference effect was not due to the variability embedded in the verbs chosen, that is, to the so-called “language as fixed effect fallacy” problem [16] (experiment 1).

Fourthly, we wanted to estimate the start and the duration of the interference effect (experiments 1, 3a and 3b). In Sato et al. [11] this effect was present only when the delay between verb presentation and the go signal was 150 ms, but not when it was set at 1150 ms. In the literature a few attempts to study the time course of the ACE effect have been reported [17]–[19]. However, the processing of single verbs is very likely to be extremely different from the processing of sentences. In fact, the ACE experiments are set up in such a way that participants could detect both the verb of the sentence, and the key words relevant for understanding the directionality of the actions, before giving a response. As a consequence participants' responses were never required earlier than 500 ms from the stimulus onset (e.g. [19]). In contrast when single verbs have been employed, it has been shown that an interaction between language processes and overt motor behavior occurs as early as 150 ms after stimulus presentation [20]–[22], [11]. These findings suggest that the cortical motor system is modulated in a period overlapping that for word understanding [23]. What remains unclear is when the effect disappears and whether it could start before 150 ms, as one study seems to suggest [12]. To further explore the timing issue from a different perspective, we also manipulated the duration of verb presentation in order to see whether it could affect the magnitude of the interference (experiment 2).

Fifthly, we wanted to assess whether the processing of action-related language is left-lateralized, in agreement with the well known lateralization of linguistic functions (e.g. [24], [25]; experiment 1). If this were the case, then when the meaning of hand-related verbs has to be understood, the RTs of reaching movements executed with the right arm should be faster than those executed with the left arm. This is a hotly debated topic since different papers report very different results (e.g. see [10], [14], [15], [26]).

Sixthly, we checked whether the interference between actions and the corresponding verbs occurs only when the semantic content of a verb has to be retrieved. To this aim we compared the performance of participants in the standard task and in a task where the same hand-, foot- and abstract verbs were presented but participants had to respond only when verbs were printed in green, and to stop when verbs were printed in red (experiment 4). Thus, in the latter task we tested whether a non-linguistic feature of an action verb could lead to the interference effect. This control experiment is crucial because, while Sato et al. [11] showed that in a lexical task the interference effect disappeared, other studies found different results (e.g. [14], [15], [26]). For instance, Scorolli and Borghi [27] found that, in a sentence comprehension task, responses were faster when the effector used for responding was the same as that involved in the action described by the sentence.

All in all our results are compatible with the theory of embodied language and provide a considerable extension of the results of Sato et al [11], thus providing a more detailed knowledge of the link between action-related language and motor acts.

## Methods

### 1.1 Subjects

Sixty-seven participants took part in the study and were rewarded with course credits. Eighteen took part in experiment 1 (mean ± SEM age: 26±0.6 years); 13 in Experiment 2 (mean age 24±0.7 years); 12 in Experiment 3a (mean age 23±0.9 years); 13 in Experiment 3b (mean age 22±0.5 years) and 12 in Experiment 4 (mean age 24±0.9 years). All participants were native Italian speakers; they were all right-handed, as assessed with the Edinburgh handedness inventory [28], and they had normal or corrected-to-normal vision and no history of language disorders. None of them was informed about the purpose of the experiments. The experimental procedures were approved by the local ethics board of the Neuromed hospital and performed in accordance with the ethical standards laid down in the 1964 Declaration of Helsinki. All subjects gave their written informed consent.

### 1.2 Verbal Stimuli

In all experiments, verbs were presented in the visual modality. We selected thirty Italian verbs in the infinitive form (see [11] and Table 1). Ten verbs referred to hand-related action (e.g. “tagliare”, “to chop”), 10 referred to foot-related action (e.g. “correre”, “to run”) and 10 referred to an abstract meaning (e.g. “scordare”, “to forget”). Verbs were matched for syllable number, word length and total lexical frequency ([29]; number of instances per ∼4,000,000 words). A one-way analysis of variance did not show significant differences between verb categories for syllable number [F(2,27) = 0.5, p = 0.61] word length [F(2,27) = 1.55, p = 0.23] or lexical frequency [F(2,27) = 0.9, p = 0.4]. In addition we asked 44 participants, who did not participate in the experiments, to rate the imageability of the verbs on a 7-point scale, where 1 indicated that the verb could not be imagined while 7 indicated that the verb was very easy to imagine. A one-way repeated-measures analysis of variance (factor: verb category) showed a main effect (F[1.03,24.6] = 15.2, p<0.001). *Post hoc* tests (pairwise comparisons with Bonferroni correction) showed that the imageability of hand- and foot-related verbs did not differ (p = 0.71) but the imageability of both verb categories was different from that of abstract verbs (both p<0.001).

**Table 1:** List of verbs used in the experiments.

	Verb	Letters	Syllables	Lexical Frequency	Imageability	Translation
**Hand-relatedverbs**	Firmare	7	3	407	6.98	to sign
	Tagliare	8	3	379	6.95	to chop
	Disegnare	9	4	190	6.93	to draw
	Applaudire	10	4	65	6.93	to applaud
	Ricamare	8	4	30	6.57	to embroider
	Timbrare	8	3	8	6.86	to stamp
	Stappare	8	3	4	6.80	to uncap
	Svitare	7	3	3	6.84	to unscrew
	Rammendare	10	4	3	6.30	to mend
	Sbottonare	10	4	2	6.80	to unbutton
	Mean(±Sem)	8.5±0.37	3.5±0.17	109.1±160.4	6.80±0.05	
**Foot-relatedverbs**	Correre	7	3	662	6.95	to run
	Camminare	9	4	234	6.98	to walk
	Marciare	8	3	45	6.68	to march
	Pedalare	8	4	37	6.89	to pedal
	Calpestare	10	4	30	6.86	to trample
	Inciampare	10	4	17	6.84	to stumble
	Zoppicare	9	4	10	6.55	to hobble
	Calciare	8	3	8	6.93	to kick
	Saltellare	10	4	6	6.95	to jump
	Pattinare	9	4	4	6.75	to skate
	Mean(±Sem)	8.8±0.33	3.7±0.15	105.3±65.6	6.84±0.04	
**Abstractverbs**	Amare	5	3	818	5.64	to love
	Temere	6	3	334	5.25	to fear
	Approvare	9	4	254	5.68	to approve
	Godere	6	3	241	5.63	to enjoy
	Sopportare	10	4	154	5.55	to bear
	Odiare	6	3	115	5.11	to hate
	Ammirare	8	4	110	5.61	to admire
	Contemplare	11	4	45	5.16	to contemplate
	Scordare	8	3	42	5.16	to forget
	Meditare	8	4	34	5.45	to meditate
	Mean(±Sem)	7.7±0.62	3.5±0.17	214.7±74.3	5.42±0.25	

For each item, number of letters, number of syllables, lexical frequency, imageability and English translation are given. Mean number of letters, syllables lexical frequency and imageability (±SEM) are reported separately for each verb category.

In Experiment 4, we employed just half of the verbs (five for each category, randomly selected). They were matched for syllable number (mean ± SEM: 3.4±0.24, 3.6±0.24 and 3.4±0.24 syllables for hand-related, foot-related and abstract verbs, respectively) and for word length (mean ± SEM: 8.4±0.51, 8.8±0.58 and 7.6±1.03 letters for hand-related, foot-related and abstract verbs, respectively). Mean lexical frequency (± SEM) for hand-, foot- and abstract-related verbs was 214.2±77.8, 197.6±122.7 and 210.8±152.5, respectively. A one-way analysis of variance did not show significant differences between verb categories for syllable number [F(2,12) = 0.2, p = 0.84], word length [F(2,12) = 0.68, p = 0.53] or lexical frequency [F(2,12) = 0.005, p = 0.99].

### 1.3 Behavioral tasks

**1.3.1** ***Experiment 1*** *(semantic task with extended presentation of verbs)*

The experiment was carried out in a sound-attenuated and dimly illuminated room. Participants sat comfortably at about 50 cm from a 17-inch PC monitor (CRT non-interlaced, refresh rate 75 Hz, 640×480 resolution, 32-bit color depth) equipped with a touch screen (MicroTouch; sampling rate 200 Hz) for touch-position monitoring. A noncommercial software package, CORTEX (http://www.cortex.salk.edu), was used to control stimulus presentation and to collect behavioral responses. The temporal arrangements of stimulus presentation were synchronized with the monitor refresh rate.

**Figure 1:**
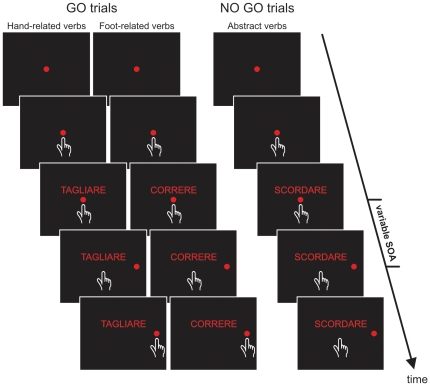
Schematic representation of experiments 1, 2, 3a and 3b. Each trial started with the presentation of a central red circle that subjects had to touch and hold for a variable period. Then, a verb was shown above the central stimulus. After a variable delay (stimulus onset asynchrony, SOA) a peripheral target appeared. Participants were asked either to touch it, if the meaning of the verb referred to a concrete action (go-trials), or to refrain from moving if it had an abstract content (no go trials; see Methods for more details).

Participants performed, in separate sessions counterbalanced across participants, the same task twice: once with the right and once with the left arm. Each trial began with the presentation of a central red circle (diameter: 3.2 degrees of visual angle [dva], or 2.8 cm) that participants had to touch with their index finger and to hold (continue touching) for a variable period (400–700 ms). Thereafter, a verb was presented just above the central circle and participants were instructed to carefully read it. When the verb referred to a concrete action (go trials) participants had to reach and hold for a variable period (300–400 ms) a peripheral red circle (3.2 dva or 2.8 cm diameter) appearing either to the right or to the left of the screen (according to the arm used) at an eccentricity of 9.1 dva (or 8 cm). Conversely, when the verb described an abstract action (no-go trials) participants had to keep the index finger still on the central stimulus for 400–800 ms (Fig. 1). Successful trials were signaled by an acoustic feedback. The go-signal, given by the presentation of the peripheral target, was delivered either 53.2 ms, (i.e. four refresh rates, RRs), after the presentation of the verb (stimulus onset asynchrony; SOA) or at an SOA of 332.5 ms (i.e. 25 RRs). We employed these two SOAs because they gave two time points around the time window within which Sato et al [11] found the interference effect. Verbs remained visible until the end of the trial. All verbs were printed in red and were presented against a dark background with uniform luminance (<0.01 cd/m^2^). Each verb was presented eight times for each SOA; thus the experiment consisted of 480 trials, run in two blocks. Verb presentation was randomized and error trials were repeated until participants completed the entire block.

**1.3.2 Experiment 2** (semantic task with brief presentation of verbs). The general procedure was identical to that described for the first experiment, except for the fact that verbs were presented just for the duration of the SOAs (53.2 and 332.5 ms). The experiment consisted of 480 trials, run in two blocks, and participants performed it only with the right arm.

**1.3.3 Experiment 3a** (time course: time window of about 400 ms). The general procedure was the same as described for the Experiment 1 with the difference that we employed the following five SOAs: 53.2, 146.3 (i.e. 11 RRs), 252.7 (i.e. 19 RRs), 345.8 (i.e. 26 RRs) and 452.2 ms (i.e. 34 RRs), covering a time window of about 400 ms. Each verb was presented six times at each SOA; thus the experiment consisted of 900 trials, run in four blocks. The experiment was performed only with the right arm.

**1.3.4 Experiment 3b** (time course: time window of about 1000 ms). The general procedure was the same as described for Experiment 3a; however, we varied the length of the five SOAs: 53.2, 332.5, 598.5 (i.e. 45 RRs), 864.5 (i.e. 65 RRs) and 1130.5 ms (i.e. 85 RRs), covering a time window of about 1 sec.

**Figure 2:**
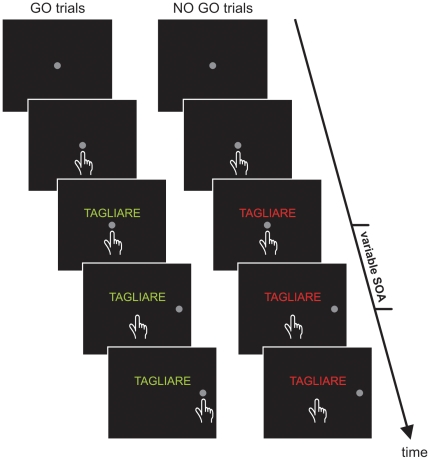
Schematic representation of the color-discrimination task (experiment 4). Each trial started with the presentation of a grey central target that participants had to touch and hold for a variable period. After a variable delay (stimulus onset asynchrony, SOA) a grey peripheral target appeared and participants were asked either to touch it if it was printed in green (go-trials) or to stay still if it was printed in red (no go trials; see Methods for more details).

**1.3.5 Experiment 4** (color discrimination task). In contrast to all the other experiments, in which participants had to move on the basis of a semantic judgment, in this experiment they were instructed to execute or refrain from their movement according to the color in which verbs were printed. Each trial started with the presentation of a central target (a grey circle with a diameter of 3.2 dva or 2.8 cm) that participants had to touch and hold for a variable period (400–700 ms). Thereafter, a verb was displayed above the central target. When the verb was printed in green, subjects were instructed to reach, as fast as possible, the peripheral target (a gray circle with a diameter of 3.2 dva or 2.8 cm) that was presented on the right side with an eccentricity of 9.1 dva (or 8 cm). Conversely, when the verb was printed in red participants had to refrain from moving (Fig. 2). Each verb was presented 12 times at each SOA; half of the times it was printed in green and in the other half it was printed in red. Thus the experiment consisted of 360 trials, run in two blocks. The experiment was performed only with the right arm.

### 1.4 Data analyses

For each participant, the mean RTs of correct trials and the mean percentages of errors were calculated for each verb category. Repeated-measures analyses of variance (ANOVA) were performed to assess differences in RTs and error rates: a) between the two arms with respect to the verb category and the two SOAs (Experiment 1); b) with respect to the verb category and the different time of verb display (Experiment 2); and c) with respect to the verb category and the different SOAs employed (Experiments 3, 4a and 4b). Mauchley's test evaluated the sphericity assumption and, where appropriate, correction of the degrees of freedom was made according to the Greenhouse–Geisser procedure. Bonferroni correction was applied to all post hoc tests (pairwise comparisons).

In addition a linear mixed model was employed to account for fixed and random effects [16], [30]. This analysis allows us to exclude the possibility that any difference between verb categories could be due to the variability embedded in the words chosen for composing the two lists of verbs instead of being a genuine effect of verb category *per se.* We considered as fixed effects the factors verb category (hand/foot), SOA (53.2 ms/332.5 ms) and, just in the case of experiment 1, arm (right/left). The factors verb (mean RT obtained at each verb) and participants were considered as random factors.

## Results

### 1.1 Experiment 1

#### 1.1.1. Interference effect: RTs and MTs

**Figure 3:**
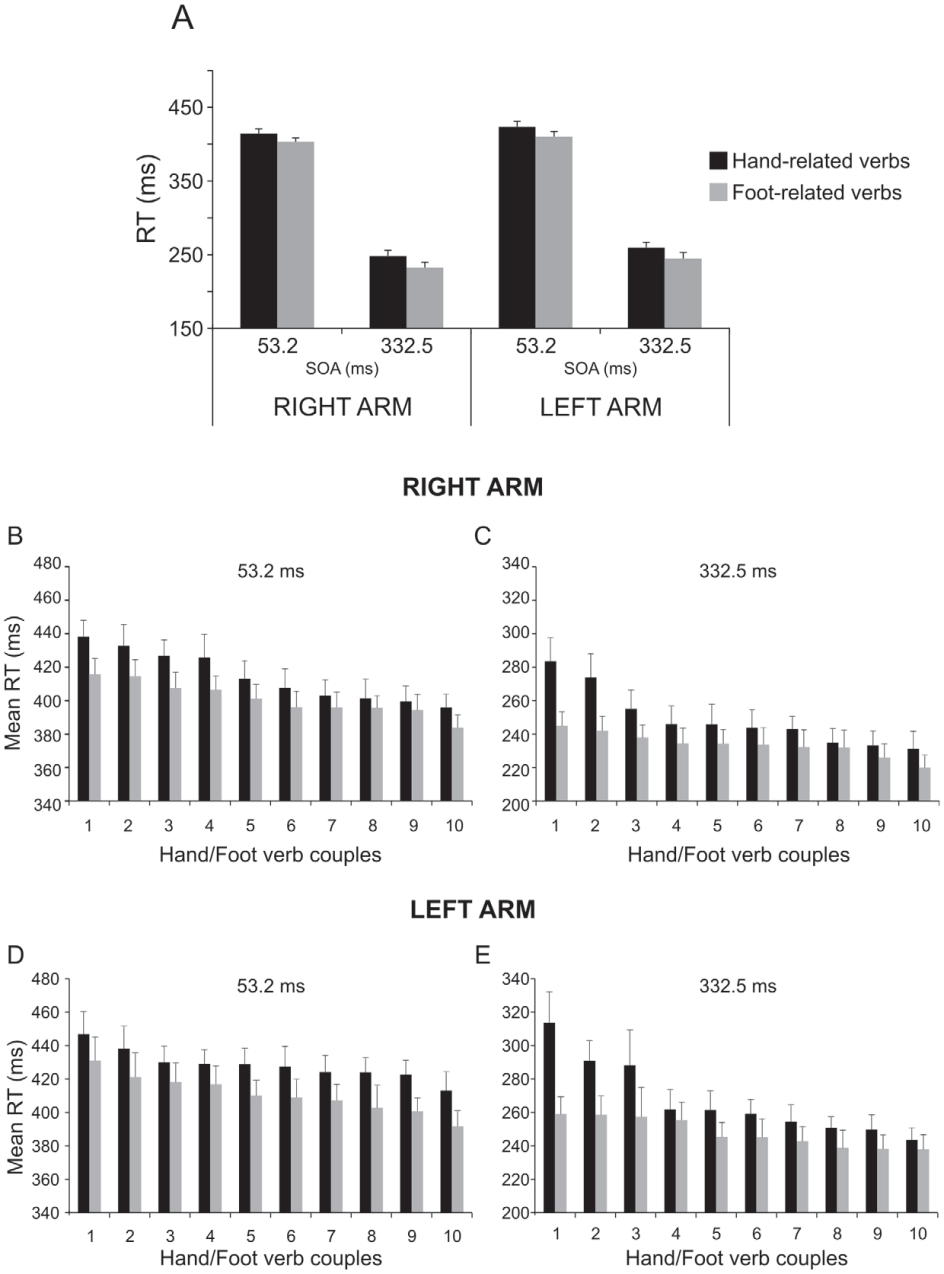
Effect of interference of verb category on reaction times (RTs) of arm reaching movements in a semantic task with extended presentation of verbs (experiment 1). Mean RT (Panel A) obtained when participants responded to hand- and foot-related verbs at the a stimulus onset asynchrony (SOA) of 53.2 ms and at an SOA of 332.5 ms either with the right and the left arm. Ranking of the mean RTs of each hand- and foot-related verb at a stimulus onset asynchrony (SOA) of 53.2 ms (Panels B and D) and at an SOA of 332.5 m at each SOA (Panels C and E) obtained when the right and the left arm were employed. The slowing of RTs for hand-related verbs with respect to foot-related verbs was present at each item. In addition, mean RTs for actions executed with the left arm tended to be slower than those executed with the right arm.

To assess the effect of verb processing on reaching arm movements, we performed a three-way repeated-measures ANOVA on RTs and on MTs, with arm (left, right), verb category (hand-related, foot-related) and SOA (short [53.2 ms], long [332.5 ms]) as factors. As far as the RTs are concerned (see fig. 3A), we found a significant main effect of factors verb [F(1,17) = 39.72, p<0.001] and SOA [F(1,17) = 984.64, p<0.001], while the factor arm was very close to being significant [F(1,17) = 3.53, p = 0.07]. RTs were significantly slower when participants responded to hand-related verbs (mean ± SEM: 336.4±5.86 ms) than to foot-related verbs (322.7±5.67 ms). Furthermore, RTs were significantly slower when the go-signal was presented after an SOA of 53.2 ms than after a SOA of 332.5 ms (412.7±5.77 ms vs. 246.3±6.7 ms).

Importantly the interference effect was present at each item (fig. 3B), suggesting that this phenomenon could not be due to the attributes of the chosen verbs (e.g. hand- or leg-verbs having the same first letters as abstract verbs might delay the response only because the decision about moving is more difficult than when the first letters are similar). To statistically assess this finding, we analyzed the RTs using a linear mixed model. In this analysis, verb category, SOA and arm were the fixed factors while verbs and participants represented the random factors. We found that all fixed factors were significant (verb category: F(1,18) = 11.1, p<0.01; SOA: F(1,1370) = 6606.3, p<0.001; arm: F(1,1370) = 48.6, p<0.001). Interactions between fixed factors were never significant. Therefore verb category, SOA and arm are good predictors of the dependent variable (the RTs). As a consequence, the differences we observed between hand- and foot-verb categories could not be ascribed to the variability embedded either in the words or in the participants selected. In contrast to the three-way ANOVA, this statistical approach revealed a highly significant effect of the factor arm indicating that subjects were overall faster with the right than with the left hand (for more information see paragraph 1.4 of the Methods section).

As far as the MTs are concerned, we found that their length was not affected by the verb presented (see Supporting Information S1).

#### 1.1.2. Interference effect: errors percentage

**Figure 4:**
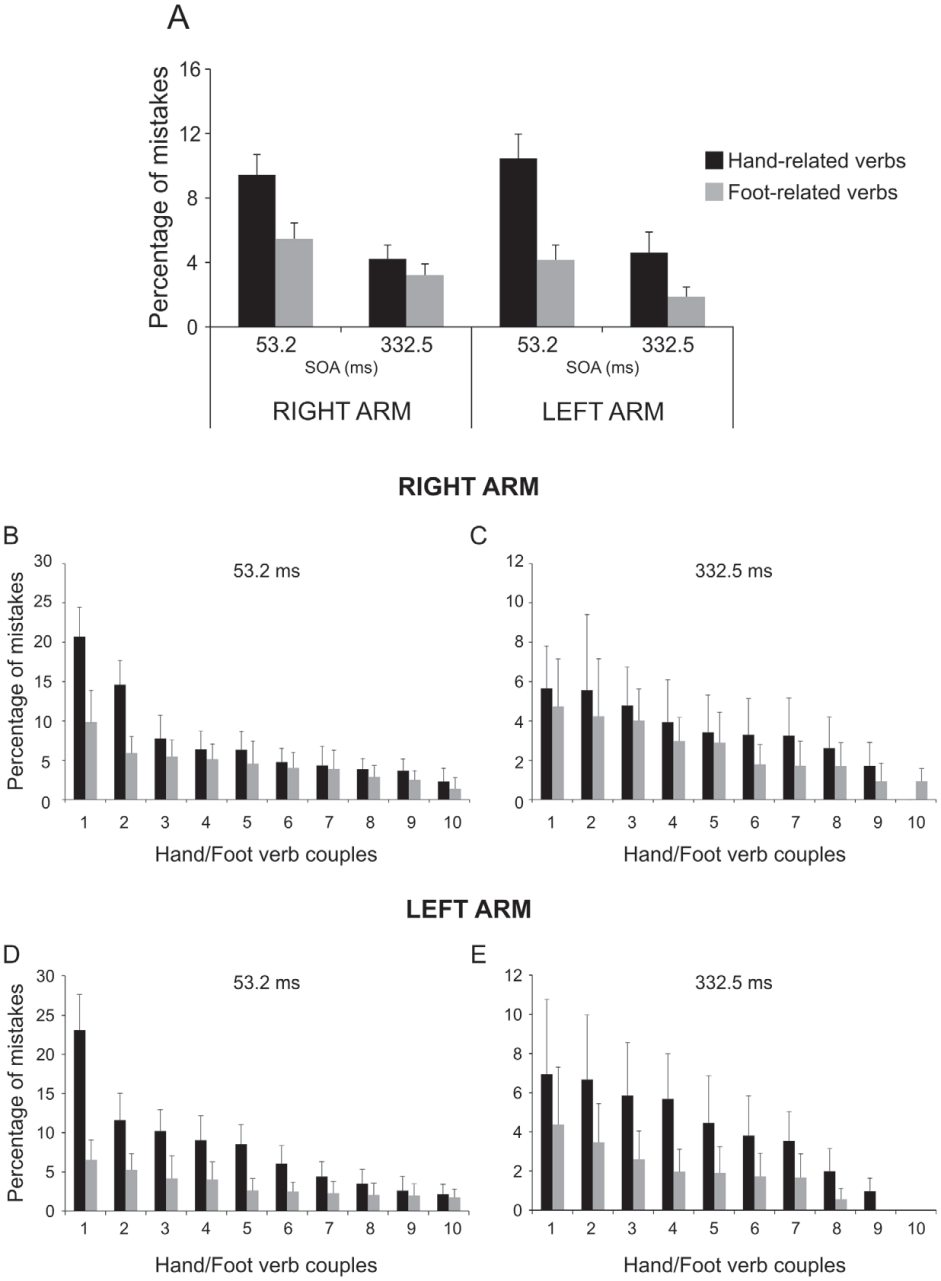
Effect of interference of verb category on error percentages of arm reaching movements in a semantic task with extended presentation of verbs (experiment 1). Mean error rates (Panel A) obtained when participants responded to hand- and foot-related verbs at the a stimulus onset asynchrony (SOA) of 53.2 ms and at an SOA of 332.5 ms either with the right and the left arm. Ranking of the mean error rates of each hand- and foot-related verb at a stimulus onset asynchrony (SOA) of 53.2 ms (Panels B and D) and at an SOA of 332.5 m at each SOA (Panels C and E) obtained when the right and the left arm were employed. Almost at each item the error percentages were higher for hand-related verbs than for foot-related verbs.

The analysis of errors (three-way repeated-measures ANOVA, factors: arm [left, right], verb category [hand-related, foot-related] and SOA [short:53.2 ms, long: 332.5 ms]; fig. 4A) showed a significant main effect of the factor verb [F(1,17) = 23.39, p<0.001] and of the factor SOA [F(1,17) = 78.68, p<0.001] but not of the factor arm [F(1,17) = 0.1, p = 0.75]. Participants made a higher rate of errors: i) when they had to move after hand-related verbs (7.18%±0.93) than after foot-related verbs (3.68%±0.52); and ii) when the go-signal was presented at a SOA of 53.2 ms (7.38%±0.8) rather than at an SOA of 332.5 ms (3.48%±0.57). In addition, there was a significant interaction between the factors verb and SOA [F(1,17) = 13.92, p<0.005]. This interaction arose because the difference in the error percentages for hand-related vs. foot-related verbs, was larger at the SOA of 53.2 ms (9.95%±1.06 vs. 4.82%±0.73, p<0.001) than at the SOA of 332 ms (4.42%±0.92 vs 2.54%±0.39, p<0.05).

As error trials were repeated until a fixed number of correct responses was obtained (see Methods), we performed an item by item analysis in order to exclude that the average error percentage might reflect the same error performed again and again on the same item. As shown in the bottom panels of fig. 4, this was not the case. The percentage of errors was constantly higher for hand- than for leg-related verbs at each item. Similarly to what was done for the RTs, we ran a linear mixed model analysis also on the error rate (fixed factors: verb category, SOA and arm; random factors: participants and verbs). We found that the factors verb category and SOA were significant (verb category: F(1,18) = 5.4, p<0.05; SOA: F(1,1392) = 39.2, p<0.001) while the factor arm was not (F(1,1392) = 0.17, p = 0.68). Participants made a higher number of errors on hand-related than on foot-related verbs (5.7% vs 3.1%, respectively) and in short SOA than in long SOA condition (5.9% vs 2.9%, respectively). Interactions between fixed factors were never significant. All in all this analysis revealed that verb category and SOA are good predictors of the dependent variable (error rate). Therefore the differences in error percentages we observed between hand- and foot-verb categories could not be ascribed to the variability embedded either in the words or in the selected participants.

#### 1.1.3. Size of interference effect

Since we found a difference between the two arms in terms of RTs, we wondered whether the size of the interference effect differed when participants used the right or the left arm. To compare its size, we employed two-way repeated-measures analysis of variance, with arm (left, right) and SOA (short [53.2 ms], long [332 ms]) as factors and the differences either of the RTs or of the error rates between hand-related and foot-related verbs as dependent variables. The ANOVA on the RTs showed that the interference effect was the same regardless of the arm used (factor arm [F(1,17) = 0.03, p = 0.87]; factor SOA [F(1,17) = 0.92, p = 0.35]; interaction [F(1,17) = 0.24, p = 0.63]). Conversely, the ANOVA on the error rates revealed that the difference in the percentage of errors was higher at the short SOA than at the long SOA (5.1%±0.87 vs 1.9%±0.83, respectively, [F(1,17) = 13.9, p<0.01]) but again there was no difference between the two arms (factor arm [F(1,17) = 2.7, p = 0.12]; interaction [F(1,17) = 0.13, p = 0.72]). In conclusion, the magnitude of the interference effect did not depend upon the arm at play.

### 1.2 Experiment 2

Since in the previous experiment verbs remained visible until the end of the trials, we wanted to assess whether the interference effect on arm movements might depend on the amount of time during which verbs were presented. To this end, we ran a two-way repeated-measures analysis of variance with verb category (hand-related, foot-related) and duration of verb presentation (53.2 ms and 332.5 ms; it has to be remarked that these times correspond to the duration of the SOAs) both on RTs and on error percentages.

**Figure 5:**
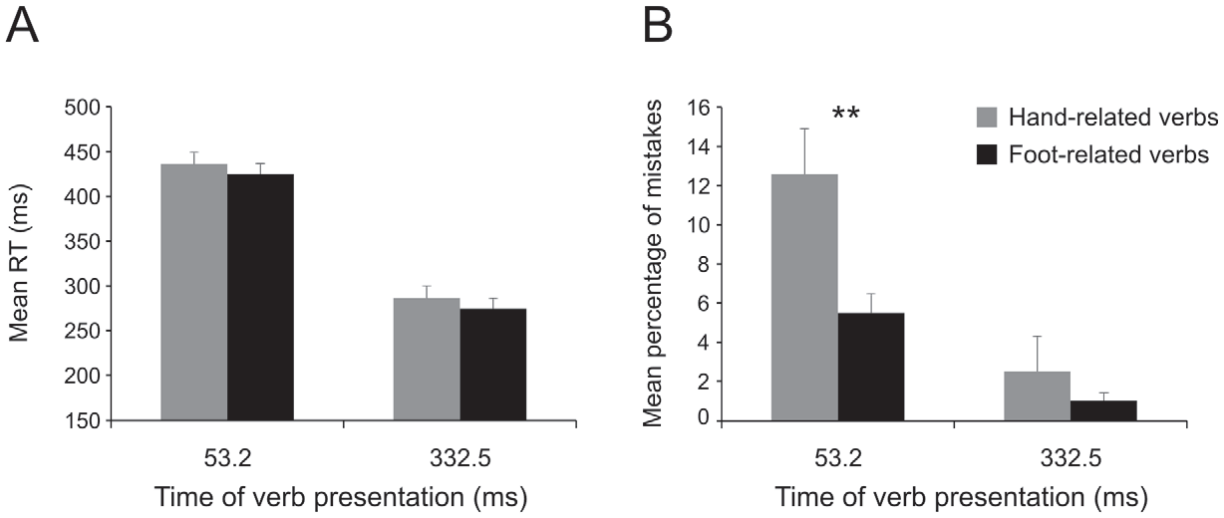
Effect of interference of verb category on arm movements in a semantic task with brief presentation of verbs (experiment 2). Mean reaction times (RT, Panel A) and mean percentage of errors (Panel B) obtained when participants responded to hand- and foot-related verbs and the verbs remained visible either for 53.2 or 332.5 ms. Movements were executed just with the right arm. ** indicates an interaction between SOA and verb category with p<0.01.

As far as the RTs are concerned (see fig. 5A), we found a significant main effect of the factor verb [F(1,11) = 17.10, p<0.005] and of the factor duration of verb presentation [F(1,11) = 2149.13, p<0.001]. The interaction was not significant [F(1,11) = 0.005 p = 0.9]. Mean RTs were significantly slower when participants responded to hand-related verbs than when they responded to foot-related verbs (361.28±11.52 vs. 349.87±10.18 ms, respectively). In addition, when verbs were presented for a brief time interval the mean RTs were significantly slower than when they were presented for a longer time (430.88±11.04 vs. 280.28±10.76 ms, respectively).

Concerning the error rates (fig. 5B), we found a main effect of the factor verb [F(1,11) = 10.21, p<0.01], of the factor duration of verb presentation [F(1,11) = 23.91, p<0.001] and a significant interaction [F(1,11) = 9.37, p<0.01]. The mean percentage of errors was higher for hand-related verbs than for foot-related verbs (7.55%±1.57 vs. 3.26%±0.9, respectively) and was also higher for short presentation than for long presentations of verbs (9.05%±1.79 vs.1.77%±0.52, respectively). Post hoc pairwise comparisons on the interaction revealed that participants made significantly more errors for hand-related verbs than for foot-related verbs only after short verb presentation (12.6%±2.3 vs.5.49±1.8, respectively, p<0.005) but not after a long verb presentation (2.51%±0.99 vs.1.03%±0.37, respectively, p = 0.19).

In order to see whether the magnitude of the interference effect was affected by the duration of verb presentation, we compared the size of the effect when verbs remained visible for the entire duration of the trials (experiment 1) with when they were on just for the duration of the SOAs (experiment 2). To evaluate the magnitude of the interference effect we looked at the difference between verb categories obtained in the two experiments in terms of both RTs and error rates. The magnitude was compared using a doubly-multivariate repeated-measures design with task (experiment 1, experiment 2) and SOAs (53.2 ms, 332.5 ms) as factors. The interference effect measured in terms of RTs had the same size in the two experiments (13.4±2.5 vs 11.4±3.3 ms; factor task [F(1,28 = 0.25, p = 0.62)]), and in the two SOAs (11.2±3 vs 13.6±2.2 ms; [F(1,28) = 0.42, p = 0.52]). The interaction was not significant [F(1,28) = 0.25, p = 0.62]), nor the percentage of errors differed between the two tasks (2.5%±0.8 vs 4.3%±1.3 [F(1,28) = 1.42, p = 0.24]), but we found a significant main effect of factor SOA [F(1,28) = 17.02, p<0.001]. In fact, participants made more errors at an SOA of 53.2 ms than at the SOA of 332.5 ms (5.55%±1.05 vs 1.24%±0.76). The interaction was not significant ([F(1,28) = 1.59, p = 0.22]).

All in all our results indicate that the interference effect did not depend on the duration of the visual presentation of verbs.

### 1.3 Experiment 3a

**Figure 6:**
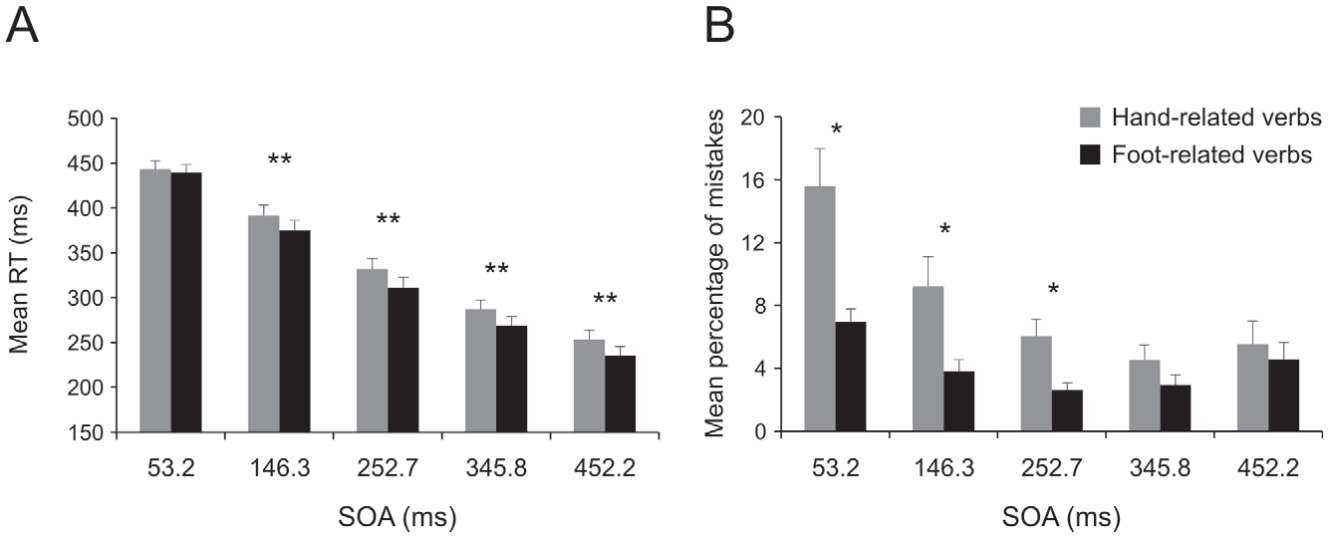
Effect of interference of verb category on arm movements in a semantic task at five different stimulus onset asynchronies (SOA) covering a time span of about 400 ms (experiment 3a). Mean reaction times (RT, Panel A) and mean percentage of errors (Panel B) obtained when participants responded to hand- and foot-related verbs at each SOA. Movements were executed just with the right arm. * indicates an interaction between SOA and verb category with p<0.05; ** p<0.01.

Given that the interference effect was still present when the SOA was set to 332.5 ms (experiment 1), we tried to assess when it disappears using progressively longer SOAs. To accomplish this, we performed a two-way repeated-measures analysis of variance with verb category (hand-related, foot-related) and SOA (SOA1–5 respectively: 53.2, 146.3, 252.7, 345.8 and 452.2 ms) as factors, on RTs and error rates separately (fig. 6A and 6B, respectively). As far as the RTs is concerned, we found a significant main effect of factor verb [F(1,11) = 119.6, p<0.001] and of factor SOA [F(4,44) = 799.4, p<0.001], and a significant interaction [F(4,44) = 3.1, p<0.05]. As expected, RTs were longer when participants had to respond to hand-related verbs than when they had to respond to foot-related verbs (341.5±10.39 vs 325.93±10.02 ms, respectively). In addition, mean RTs were longer at the shortest SOA (SOA1: RT 441.44±8.97 ms) and became gradually faster as duration of SOAs increased (SOA2: RT 383.28±11.54; SOA3: 321.54±11.37; SOA4: 277.93±10.31; SOA5: 244.39±10.03 ms). Post hoc pairwise comparisons of the interaction revealed that the mean RTs to hand-related verbs were always slower than to foot-related verbs at all SOAs (p<0.001), except from the first one (53.2 ms; p = 0.416).

As far as the error rate is concerned, we found a significant main effect of verb [F(1,11) = 15.1, p<0.005] and of SOAs [F(4,44) = 13.1, p<0.001], and an interaction [F(4,44) = 4.25, p<0.005]. The percentage of errors in response to hand-related verbs was significantly higher than that in response to foot-related verbs (8.18%±1.24 vs 4.17%±0.54, respectively). Furthermore, participants made a higher number of errors at the shortest SOA (11.29±1.5%). Post hoc pairwise comparisons showed that the percentage of errors at the first SOA was significantly higher than for all the following SOAs (all p<0.01). Post hoc pairwise comparisons of the interaction revealed that participants made more errors on hand-related verbs than on foot-related verbs at the first three SOAs (all p<0.05), that is, until 252.7 ms.

These analyses revealed that the interference effect was not over at the longest SOA (452.2 ms). This was true both for the RTs and in terms of errors. In fact, even though the percentage of errors was not significant at the last two SOAs, fig. 6 shows that the trend was not different from that of the previous SOAs.

### 1.4 Experiment 3b

**Figure 7:**
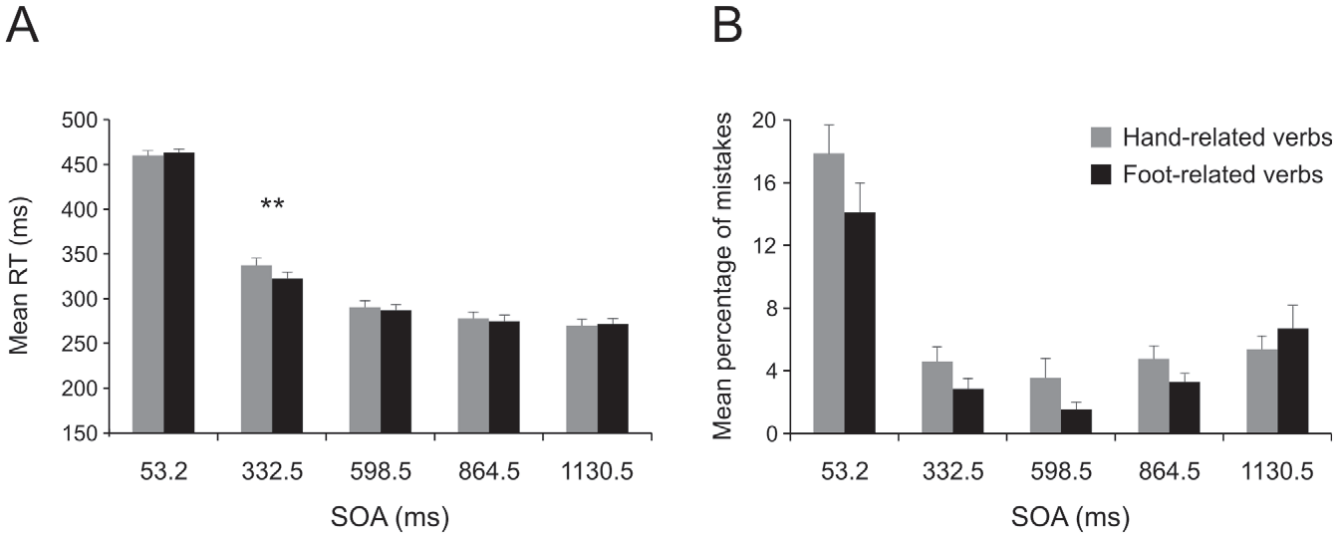
Effect of interference of verb category on arm movements in a semantic task at five different stimulus onset asynchronies (SOA) covering a time span of about 1000 ms (experiment 3b). Mean reaction times (RT, Panel A) and mean percentage of errors (Panel B) obtained when participants responded to hand- and foot-related verbs at each SOA for movements executed with the right arm. ** indicates an interaction between SOA and verb category with p<0.01.

To find out the time point at which the interference effect disappears, we repeated the previous experiment using SOAs covering a time span of 1000 ms. Again we employed a two-way repeated-measures analysis of variance with verb category (hand-related, foot-related) and SOA (SOA1–5 respectively: 53.2, 332.5, 598.5, 864.5 and 1130.5 ms) as factors, using RTs and error rates as dependent variables (fig. 7A and 7B, respectively).

The ANOVA on RTs revealed a significant main effect of the factor verb [F(1,12) = 9.95, p<0.01] and of the factor SOA [F(2.1,25.8) = 780.64, p<0.001], and a significant interaction [F(4,48) = 8.85, p<0.001]. As in the previous experiment, participants were slower when they responded to hand-related verbs than to foot-related verbs (327.12±6.25 vs 323.76±5.73 ms, respectively) but the magnitude of the effect was greatly reduced. Again the RTs became gradually faster as duration of the SOAs increased (SOA1: RT 461.8±4.59 ms; SOA2: 329.78±7.5 ms; SOA3: 288.7±6.66 ms; SOA4: 276.24±6.80 ms; SOA5: 270.67±6.55 ms). Post hoc analysis on the interaction showed that a significant difference between the two verb categories occurred only at the second SOA (332.5 ms; p<0.001).

With regard to error rate, we found a significant main effect of factor verb [F(1,12) = 7.16, p<0.02] and of factor SOA [F(2.09,25.07) = 42.21, p<0.001] but no interaction [F (4,48) = 2.04, p = 0.1]. Errors were more frequent for hand-related verbs than for foot-related verbs (7.22%±0.73 vs 5.68%±0.63, respectively). In addition, participants made the highest number of errors at the shortest SOA (15.99%±1.61; post-hoc pairwise comparisons, all p<0.001).

Overall, putting together the results of experiments 3a and 3b, we conclude that the interference effect ends at some point between 452.2 and 598.5 ms.

### 1.5 Experiment 4

**Figure 8:**
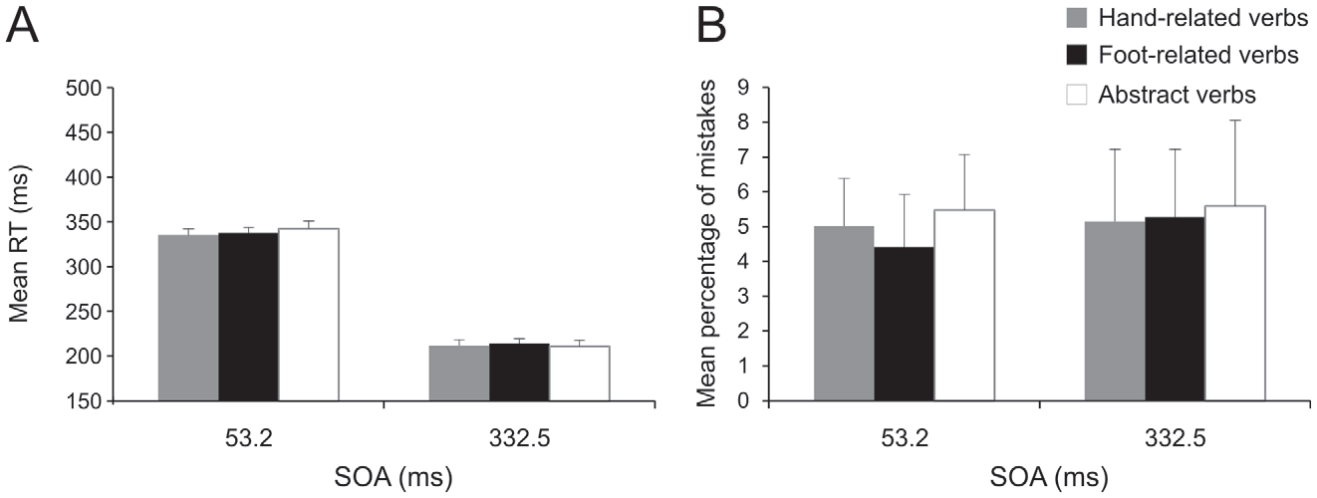
Effect of interference of verb category on arm movements in the color discrimination task (experiment 4). Mean reaction times (RT, Panel A) and mean percentage of errors (Panel B) obtained when participants responded to hand-related, foot-related and abstract (open bars) verbs at the stimulus onset asynchrony (SOA) of 53.2 ms and of 332.5 ms. Movements were executed just with the right arm.

In order to check whether the interference between action and the corresponding verb occurs only when the semantic content of a verb has to be retrieved, we ran a task in which participants had to move their arm according to the color in which the verb was printed. To compare the performance of participants during the color discrimination task in terms of either RTs or error percentages, we performed a two-way repeated-measures analysis of variance, with verb (hand-related, foot-related, abstract) and SOA (53.2 ms, 332.5 ms) as factors. The analysis of the RTs shown that there was no difference according to the verb category (factor verb [F(2,22) = 0.89, p = 0.42]; see fig. 8A). We found a main effect of factor SOA [F(1,11) = 629.26, p<0.001], indicating that participants were slower when the go-signal was delivered after 53.2 ms than when it was given after 332.5 ms (338.57±8.09 vs. 212.19±8.8 ms, respectively). The interaction was not significant [F(2,22) = 1.32, p = 0.29]. The error rate (fig. 8B) did not change (factor verb [F(2,22) = 0.96, p = 0.9]; factor SOA [F(1,11) = 0.12, p = 0.74]; interaction [F(2,22) = 0.033, p = 0.97]).

To account for item variability, we ran a linear mixed model analysis both on RTs and on error rates as for experiment 1, with verb category (hand, foot, abstract) and SOA (53.2 ms, 332.5 ms) as fixed factors and verbs and participants as random factors. As far as the RTs were concerned, we found that only the factor SOA was significant (F(1,317) = 2095.2, p<0.001), while neither the factor verb category (F(2,12) = 0.5, p = 0.63) nor the interactions between SOA and verb category reached the significance (F(2,12) = 0.5, p = 0.63). As far as the error rates were concerned, none of the fixed factors (verb category: F(2,12) = 0.12, p = 0.88; SOA: F(1,320) = 0.18, p<0.67) or the interaction were significant. These results show that the absence of differences between RTs and error percentages for different verb categories occurring when verb semantic was not the cue for movement execution did not depend on participants or on the list of chosen verbs.

## Discussion

### 1.1 How does action language affect motor responses?

It has been shown that the interaction between motor-related language and motor responses can take different directions according to the task at play (lexical versus semantic tasks) or to the type of linguistic stimuli employed (single verbs versus sentences).

A number of studies have shown that, in comprehension tasks, responses to meaningful sentences expressing a movement toward or away from the body were performed more rapidly when subjects made a movement in the same direction as that described in the sentence (e.g., [10], [19], [31]–[33]). A facilitation was also found by Scorolli and Borghi [27] again in a comprehension task, where participants responded to a meaningful sentence, which did not imply any movement directions but that described the effector used to respond. However, Buccino et al [9] in a task where participants were required to semantically process sentences in order to discriminate between action-related sentences and abstract content sentences, found exactly the opposite result, that is, they found an interference when the effector used for responding was the same as that appearing in the sentence.

These data suggest that a key variable for predicting the effect of language processing on action execution is given by the task requests. In fact, similar action-related language materials produce different outputs according to the task rules.

Findings from studies using single verbs seem to sustain this conclusion. Sato et al [11] showed that, when participants had to understand the meaning of a verb, a slowdown of the movement occurred when the action expressed by the verb involved the same effector used to give the response. In contrast, this interference did not take place in a lexical decision task, in which subjects were required to judge whether or not the stimulus was a meaningful word. However, lexical tasks do not always unequivocally provide the same results. For instance, Pulvermuller et al. [26] found that transcranial magnetic stimulation of the arm representation in M1 led to faster responses when hand-related verbs were presented than when leg-related verbs were presented (see also [14], [15]). These incongruences might be explained by the fact that both lexical tasks and passive reading tasks are based upon linguistic rules.

To further explore the nature of the interaction between single verb and motor responses, first of all we ran a semantic task inspired by the experiment of Sato et al [11]. As expected, we found that a very consistent interference effect occurred when participants had to respond with a reaching movement to an hand-related verb. Furthermore, for the first time, we showed that the interference was not limited to the RTs, it extended to the error rate but not to the MTs. This latter finding might be due either to the greater variability of MTs than of the other two variables or to the fact that neural processes occurring during movement execution are different from those occurring during movement planning.

Importantly, thanks to our statistical approach, we were able to exclude the possibility that this phenomenon could be simply due to the random variability of the chosen verbs. Secondly we demonstrated that when using non-linguistic cues, that is, presenting the same verbs used in the semantic task but asking participants to respond or to withhold their movements according to the color in which verbs were printed, the interference effect was completely wiped out.

At the very least this result indicates that, when a semantic judgment is not required, the difference between hand and foot verbs does not take place. By stating this we do not exclude the possibility that in the color discrimination task the verbs can be read, but in this instance the semantic information is simply not required to solve the task. In our opinion, this finding represents indirect evidence of the involvement of the cortical motor system in action-word understanding. The interference would occur only when the motor cortex is needed both for interpreting the verb semantic, possibly by activating the motor representation associated with that verb, and also for preparing a movement using the effector described by the verb.

We acknowledge that this finding does not indubitably lead to the conclusion that the modulation of the motor cortex is a necessary step for language understanding [11]. It could be that the motor system is engaged only as a byproduct when a subject activates the symbolic representation of action-verb meanings (see [13]). However, evidence showing that processing of action-verb information depends on the integrity of the motor system [15], [34], [35] and studies demonstrating that somatotopic magnetic stimulation of the motor system specifically influence the processing either of action words [26] or of sentences describing actions [9], lead us to hypothesize that the motor system is very likely to play a role in understanding the meaning of an action word. Obviously we do not exclude the possibility that other brain regions, i.e. other language-related brain regions, participate in the processing of action-related words. In fact, it is plausible that action words semantic could be processed by a distributed network of cortical areas encompassing non motor and motor regions [36].

The overall amplitude of the interference effect, both in terms of RTs and error rates, is relatively small. We believe that the phenomenon we observed represents a cost, related to the way in which the neural network subserving action-language processing is organized. Clearly, this cost cannot be too high, otherwise it would compromise our ability to react efficiently in presence of action-language material.

### 1.2 Time course of the interference effect

To the best of our knowledge the time course of the recruitment of cortical motor areas for single action-verbs is largely unknown. Electrophysiological studies showed that, in a lexical task, differences between face, leg and hand verb categories occur around 150 ms after stimulus presentation [20]–[22]. Sato et al. [11] demonstrated that, in a semantic decision task, the effect of verb category was present at an SOA of 150 ms, but not at 1150 ms. Recently Boulenger et al. [13] demonstrated that masked words describing motor actions activate cortical motor regions and alter the execution of subsequent reaching movements. This finding suggests that even a subconscious perception of action verbs affects motor responses. Given this, we hypothesized that the interference effect could start before 150 ms. In fact, in experiment 1 and 2, we found a significant interference both in terms of RTs and error rate when the delay between the verb presentation and the go-signal was as little as 53.2 ms (irrespective of whether the verb remained on the screen or disappeared). This result might indicate that the recruitment of the motor system occurs very early, even before the time thought to be sufficient to recruit frontal areas during reading [21], [22] or word recognition [23]. This is not unexpected because evidence from fMRI studies [37] and intracerebral electroencephalographic recordings [38] shows that subliminally presented words automatically pre-activate essential parts of the cerebral networks recruited by language processing. As a consequence, verb processing could take place quickly and automatically in some sector of the cortical motor system.

Since the interference effect did not end when the SOA was 332.5 ms, to find out its finishing point we varied the duration of the SOA from 53.2 ms to 1130.5 ms, thus covering a time span of about one second. In the experiments in which more than two SOAs were employed, the interference effect in terms of RTs appeared around 150 ms and ended at some point between 450 and 600 ms. The pattern of error rates did not completely overlap with that of RTs even though, overall, participants made more errors for hand-related than for foot-related verbs. At the shortest SOA (52.3 ms) the error rates were significantly higher for hand-related than for foot-related verbs but there was no difference in terms of RT. This fails to replicate the results obtained when only two SOAs were utilized. However, this discrepancy could be ascribed to the context in which participants operated. Indeed, the average RT at the SOA of 53.2 ms increased when five SOAs were utilized, with respect to when only two SOAs were employed, reflecting a higher degree of difficulty in the former case. Likely, under these conditions, RTs cannot be further modulated (i.e. RTs for hand-related verbs cannot increase anymore). As a consequence, the interference effect can manifest itself only in terms of error rates. In fact, at the SOA of 53.2 ms, the error rates of experiments 3a and 3b, are higher than those of experiment 1 and 2 (respectively, 15.6%±2.4 and 17.9%±1.8 versus 9.45%±1.24 and 12.6%±2.3).

In conclusion we firmly believe that the interference effect was present in experiments 3a and 3b and therefore our data indicate that, at least in our experimental context, action words influence motor responses until about 500–600 ms. Since we did not use SOAs below 53.2 ms, we could not establish a lower boundary of the interference effect. This issue will deserve further studies.

### 1.3 Lateralization of action verb processing

Several neuroimaging studies have shown that, in right-handers, activity in cortical motor areas associated with action-related words tends to be left-lateralized [5]–[7], [39]. However, patients with right frontal lesions shows a specific impairment in processing action verbs while performing a lexical task [15], indicating that these areas play a role in action-related language processing. Willems et al. [14], by comparing the activity elicited by single action-verbs in right- and left-handed subjects, during a lexical task with fMRI, found that each group preferentially activated premotor areas in the hemisphere contralateral to the dominant hand. Thus they concluded that processing of action-verb meaning is differently lateralized according to the manual preference. In contrast, Glenberg and Kaschak [10] showed that an action-sentence compatibility effect arises when participants respond with either the dominant or the non-dominant hand. The discrepancy with the findings of Willems et al. [14] could be explained by taking into account the different elaboration required to understand sentences as opposed to single verbs. Furthermore, Glenberg and Kaschak [10] compared the performance of two different groups of participants, one using the right and one using the left hand. As a consequence their results might be affected by the random variability of the two samples.

In order to get around this problem we directly compared the performance of the same participants using the left and the right arm in a task requiring them to understand the meaning of a verb. In the context of such an experiment a straightforward prediction can be tested. If the meaning of an action-related verb is understood by means of the left hemisphere, it follows that when participants have to respond with the right arm they should be faster than when responding with the left arm independently from the verb category. In fact, participants were constantly faster with the right than with the left arm, even though the size of the interference effect was not different between the two arms. We hypothesized that the interference effects would come from the recruitment of the same cortical territory in two tasks, linguistic processing and movement programming/execution. This overlap occurs when both arms are used, because the primary motor cortex and the pre-motor cortex are activated bilaterally during the production of reaching movements (e.g., [40], [41]). Such ipsilateral activation is more frequently observed when movements are performed with the non-dominant hand [42]. As a consequence when subjects move the dominant arm, i.e. the right one, the left hemisphere is activated for both linguistic elaboration and movement planning. When the left arm is employed, both the right and the left hemispheres are engaged for moving. Even though the neurophysiological significance of this phenomenon remains unclear, it has been suggested that ipsilateral activation during non-dominant hand movements could reflect an increased inhibition exerted by the right over the left hemisphere through callosal fibers [43]. This inhibition might slow down the elaboration of verb semantics occurring in the left hemisphere. Since this is a necessary step to generate an appropriate motor plan for left arm movements, the slowing down of the linguistic process causes a generalized delay of the RTs, without affecting the interference effect. The fact that right-handers are faster when reaching for a peripheral target with the right hand is not obvious. In fact, it has been shown that when right-handed persons were asked to perform reaching movements toward peripheral targets, they were systematically faster when they have to reach a left target with the left hand than when they have to reach a right target with the right hand [44]–[46]. This left hand advantage has been interpreted as reflecting a greater degree of engagement of the right hemisphere in spatial processing. Since we also required subjects to perform reaching movements toward targets ipsilateral to the arm, but we found the opposite results in term of response speed, we believe that our data indicate that verb processing in right-handers is lateralized to the left hemisphere.

## Supporting Information

Supplementary Information S1 **Analyses of movement times across all experiments.**
(DOC)

## References

[1] Fischer MH, Zwaan RA (2008). Embodied language: a review of the role of the motor system in language comprehension.. Q J Exp Psychol (Colchester).

[2] Pulvermuller F (2002). The neuroscience of language.

[3] Gallese V, Lakoff G (2005). The Brain's concepts: the role of the Sensory-motor system in conceptual knowledge.. Cogn Neuropsychol.

[4] Gallese V (2008). Mirror neurons and the social nature of language: the neural exploitation hypothesis.. Soc Neurosci.

[5] Hauk O, Johnsrude I, Pulvermuller F (2004). Somatotopic representation of action words in human motor and premotor cortex.. Neuron.

[6] Tettamanti M, Buccino G, Saccuman MC, Gallese V, Danna M (2005). Listening to action-related sentences activates fronto-parietal motor circuits.. J Cogn Neurosci.

[7] Aziz-Zadeh L, Wilson SM, Rizzolatti G, Iacoboni M (2006). Congruent embodied representations for visually presented actions and linguistic phrases describing actions.. Curr Biol.

[8] Boulenger V, Hauk O, Pulvermuller F (2009). Grasping ideas with the motor system: semantic somatotopy in idiom comprehension.. Cereb Cortex.

[9] Buccino G, Riggio L, Melli G, Binkofski F, Gallese V (2005). Listening to action-related sentences modulates the activity of the motor system: a combined TMS and behavioral study.. Brain Res Cogn Brain Res.

[10] Glenberg AM, Kaschak MP (2002). Grounding language in action.. Psychon Bull Rev.

[11] Sato M, Mengarelli M, Riggio L, Gallese V, Buccino G (2008). Task related modulation of the motor system during language processing.. Brain Lang.

[12] Boulenger V, Silber BY, Roy AC, Paulignan Y, Jeannerod M (2008). Subliminal display of action words interferes with motor planning: a combined EEG and kinematic study.. J Physiol Paris.

[13] Mahon BZ, Caramazza A (2005). The orchestration of the sensory-motor systems: Clues from Neuropsychology.. Cogn Neuropsychol.

[14] Willems RM, Hagoort P, Casasanto D (2010). Body-specific representations of action verbs: neural evidence from right- and left-handers.. Psychol Sci.

[15] Neininger B, Pulvermuller F (2003). Word-category specific deficits after lesions in the right hemisphere.. Neuropsychologia.

[16] Clark HH (1973). The language-as-fixed effect fallacy: A critique of language statistics in psychological research.. Journal of Verbal Learning and Behavior.

[17] Zwaan RA, Taylor LJ (2006). Seeing, acting, understanding: motor resonance in language comprehension.. J Exp Psychol Gen.

[18] Bub DN, Masson ME (2010). On the nature of hand-action representations evoked during written sentence comprehension.. Cognition.

[19] Kaschak MP, Borreggine KL (2008). Temporal dynamics of the action-sentence compatibility effect.. Q J Exp Psychol (Colchester ).

[20] Boulenger V, Roy AC, Paulignan Y, Deprez V, Jeannerod M (2006). Cross-talk between language processes and overt motor behavior in the first 200 msec of processing.. J Cogn Neurosci.

[21] Hauk O, Pulvermuller F (2004). Neurophysiological distinction of action words in the fronto-central cortex.. Hum Brain Mapp.

[22] Hauk O, Shtyrov Y, Pulvermuller F (2008). The time course of action and action-word comprehension in the human brain as revealed by neurophysiology.. J Physiol Paris.

[23] Hauk O, Davis MH, Ford M, Pulvermuller F, Marslen-Wilson WD (2006). The time course of visual word recognition as revealed by linear regression analysis of ERP data.. Neuroimage.

[24] Ojemann GA (1991). Cortical organization of language.. J Neurosci.

[25] Galaburda AM, LeMay M, Kemper TL, Geschwind N (1978). Right-left asymmetrics in the brain.. Science.

[26] Pulvermuller F, Hauk O, Nikulin VV, Ilmoniemi RJ (2005). Functional links between motor and language systems.. Eur J Neurosci.

[27] Scorolli C, Borghi AM (2007). Sentence comprehension and action: effector specific modulation of the motor system.. Brain Res.

[28] Oldfield RC (1971). The assessment and analysis of handedness: the Edinburgh inventory.. Neuropsychologia.

[29] Bertinetto PM, Burani C, Laudanna A, Marconi L, Ratti D (2005).

[30] Raaijmakers JG (2003). A further look at the “language-as-fixed-effect fallacy”.. Can J Exp Psychol.

[31] Borreggine KL, Kaschak MP (2006). The Action-Sentence Compatibility Effect: It's All in the Timing.. Cogn Sci.

[32] Zwaan RA, Stanfield RA, Yaxley RH (2002). Language comprehenders mentally represent the shapes of objects.. Psychol Sci.

[33] Aravena P, Hurtado E, Riveros R, Cardona JF, Manes F (2010). Applauding with closed hands: neural signature of action-sentence compatibility effects.. PLoS One.

[34] Boulenger V, Mechtouff L, Thobois S, Broussolle E, Jeannerod M (2008). Word processing in Parkinson's disease is impaired for action verbs but not for concrete nouns.. Neuropsychologia.

[35] Bak TH, O'Donovan DG, Xuereb JH, Boniface S, Hodges JR (2001). Selective impairment of verb processing associated with pathological changes in Brodmann areas 44 and 45 in the motor neurone disease-dementiaaphasia syndrome.. Brain.

[36] Pulvermüller F (2005). Brain mechanisms linking language and action.. Nat Rev Neurosci.

[37] Dehaene S, Naccache L, Cohen L, Bihan DL, Mangin JF (2001). Cerebral mechanisms of word masking and unconscious repetition priming.. Nat Neurosci.

[38] Naccache L, Gaillard R, Adam C, Hasboun D, Clemenceau S (2005). A direct intracranial record of emotions evoked by subliminal words.. Proc Natl Acad Sci U S A.

[39] Desai RH, Binder JR, Conant LL, Seidenberg MS (2010). Activation of sensory-motor areas in sentence comprehension.. Cereb Cortex.

[40] Cisek P, Crammond DJ, Kalaska JF (2003). Neural activity in primary motor and dorsal premotor cortex in reaching tasks with the contralateral versus ipsilateral arm.. J Neurophysiol.

[41] Donchin O, Gribova A, Steinberg O, Mitz AR, Bergman H (2002). Single-unit activity related to bimanual arm movements in the primary and supplementary motor cortices.. J Neurophysiol.

[42] Kawashima R, Matsumura M, Sadato N, Naito E, Waki A (1998). Regional cerebral blood flow changes in human brain related to ipsilateral and contralateral complex hand movements a PET study.. Eur J Neurosci.

[43] Kobayashi M, Hutchinson S, Schlaug G, Pascual-Leone A (2003). Ipsilateral motor cortex activation on functional magnetic resonance imaging during unilateral hand movements is related to interhemispheric interactions.. Neuro Image.

[44] Barthelemy S, Boulinguez P (2002). Manual asymmetries in the directional coding of reaching: further evidence for hemispatial effects and right hemisphere dominance for movement planning.. Exp Brain Res.

[45] Barthelemy S, Boulinguez P (2001). Manual reaction time asymmetries in human subjects: the role of movement planning and attention.. Neurosci Lett.

[46] Boulinguez P, Nougier V, Velay JL (2001). Manual asymmetries in reaching movement control. I: Study of right-handers.. Cortex.

