# Supplementary material for: Processing of Hand-Related Verbs Specifically Affects the Planning and Execution of Arm Reaching Movements
Source: PLoS One. 2012 Apr 20;7(4):e35403. doi: 10.1371/journal.pone.0035403 (PMC3335064; doi:10.1371/journal.pone.0035403)
Supplement: Supplementary Information S1 — Analyses of movement times across all experiments. (DOC) [file pone.0035403.s001.doc]

**SUPPLEMENTARY MATERIALS**

1. **Interference effect and Movement Times (MTs)**
   1. *Experiment 1 (semantic task with longly presented verbs)*

Results are shown in Table 1 and Fig 1. To assess the effect of verb processing on MTs, we ran a 3-way repeated measures ANOVA with verb (hand, foot), stimulus onset asynchrony (SOA: 53.2, 332.5 ms) and arm (right, left) as factors. We found a significant main effect of SOA [F(1,17)=7.51, p<0.05] as participants made faster movements toward the peripheral target when it was delivered at 53.2 ms than at 332.5 ms (268.9±12.5 vs.. 282.9±11.9, respectively). Furthermore, we found a significant interaction between verb and SOA [F(1,17)=5.88, p<0.05]. The interaction was due to the fact that: a) at the longest SOA, hand-related verbs were significantly faster than foot-related verbs (280.7±11.7 vs.. 285.1±12.3 ms, respectively); b) at the shortest SOA foot-related verbs were significantly faster than at the longest SOA (266.9±12.6 vs.. 285.1±12.3, respectively). Neither the factor arm [F(1,17)=2.49, p=0.13] or the factor verb category [F(1,17)=0.007, p=0.94] were significant.

In conclusion, differently from what we obtained for the reaction times (RTs), we did not find a main effect of the factor verb on MTs, because at the two SOAs hand-related and foot-related verbs behaved differently.

|  |  | **EXP 1** | |  | **EXP 2** |  | **EXP 4** |
| --- | --- | --- | --- | --- | --- | --- | --- |
|  |  | RIGHT ARM | LEFT ARM |  |  |  |  |
| **HAND** | **53.2** | 262.92±12.9 | 278.76±13.8 |  | 299.12±9.3 |  | 302.37±17.7 |
|  | **332.5** | 273.39±13.1 | 288.15±11.8 |  | 306.37±11.9 |  | 317.24±19.5 |
| **FOOT** | **53.2** | 258.32±14.1 | 275.54±12.7 |  | 290.62±10.6 |  | 308.54±18.7 |
|  | **332.5** | 279.87±13.2 | 290.15±12.7 |  | 306.66±12.1 |  | 319.65±20.5 |
| **ABSTRACT** | **53.2** | - | - |  | - |  | 309.05±21.3 |
|  | **332.5** | - | - |  | - |  | 321.64±10.2 |

Table 1 Movement times (±SEM) recorded during Experiment 1, Experiment 2 and Experiment 4. For each experiment, movement times for hand-, foot- (Exp1 and 2) and abstract-verbs (Exp 4) are reported for both short (53.2 ms) and long stimulus onset asynchrony (332.5 ms).


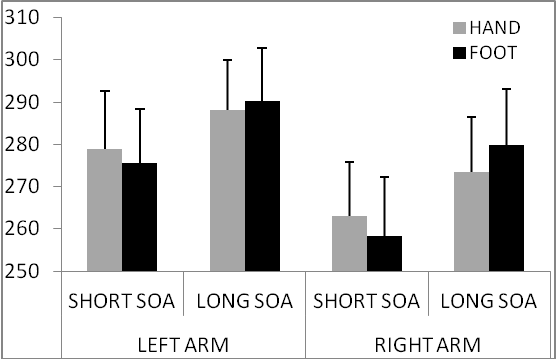


**Fig 1. Effect of interference of verb category on arm movements in a semantic task with long presented verbs (experiment 1).** Mean movement times obtained when participants responded to hand- and foot-related verbs at the a stimulus onset asynchrony (SOA) of 53.2 ms and at a SOA of 332.5 ms. Movements were executed both with the left and the right arm.

- 1. *Experiment 2 (semantic task with shortly presented verbs)*

Results are shown in Table 1 and Fig 2. To assess the effect of verb processing on MTs when verbs were presented for a short time, we ran a 2-way repeated measures ANOVA with verb (hand, foot) and duration of verb presentation (53.2, 332.5 ms) as factor. We found only a significant main effect of SOA [F(1,11)=15.7, p<0.01], because subjects were significantly faster when the verb was presented for 53.2 ms than when it was presented for 332.5 ms (294.9±9.8 vs. 306.5±11.9, respectively). Neither the factor verb [F(1,11)=3.9, p=0.07] or the interaction [F(1,11)=2.6, p=0.13] were significant.

In conclusion, differently from what we obtained for the RTs, in this experiment MTs do not seem to be affected by verb processing at all.


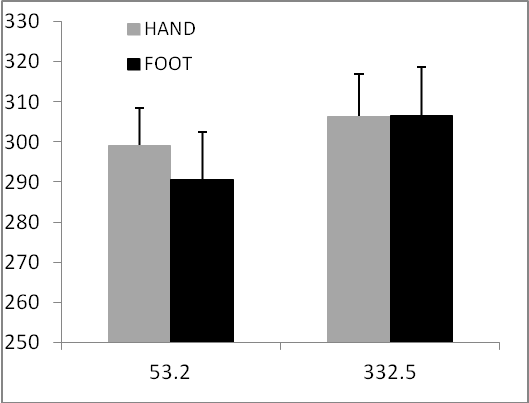


Fig 2. **Effect of interference of verb category on arm movements in a semantic task with shortly presented verbs (experiment 2)**. Mean movement times obtained when participants responded to hand- and foot-related verbs and the verbs remained visible either for 53.2 or 332.5 ms. Movements were executed just with the right arm.

- 1. *Experiment 3a (time course: time window of about 400 ms)*

Results are shown in Table 2 and in fig. 3. To assess the effect of verb processing on MTs at different SOAs, we ran a 2-way repeated measures ANOVA with verb (hand, foot) and SOA (53.2, 146.3, 252.7, 345.8, 452.2) as factors. We found a significant main effect of factor SOA [F(4,44)=4.41, p<0.01], however post-hoc pairwise comparisons did not show any significant difference after the Bonferroni correction for multiple comparisons. Neither the factor verb [F(1,11)=2.35, p=0.15] or the interaction [F(4,44)=0.73, p=0.58] were significant.

In conclusion, differently from what we obtained for the RTs, in this experiment MTs do not seem to be affected by verb processing at all.

|  |  | **HAND** |  |  | **FOOT** |
| --- | --- | --- | --- | --- | --- |
| **EXP 3a** | **53.2** | 315.51±17.1 |  |  | 313.69±17.4 |
|  | **146.3** | 316.52±16.5 |  |  | 318.74±19.1 |
|  | **252.7** | 313.88±16.9 |  |  | 319.22±16.2 |
|  | **345.8** | 320.57±15.8 |  |  | 321.50±16.9 |
|  | **452.2** | 323.94±17.3 |  |  | 327.78±17.8 |
| **EXP 3b** | **53.2** | 290.06±14.7 |  |  | 276.80±16.1 |
|  | **332.5** | 297.63±15.1 |  |  | 297.04±15.6 |
|  | **598.5** | 306.74±14.9 |  |  | 308.60±14.2 |
|  | **864.5** | 309.68±13.3 |  |  | 309.36±14.1 |
|  | **1130.5** | 306.96±13.8 |  |  | 305.55±13.6 |

Table 2. Movement times (±SEM) recorded during Experiment 3a and Experiment 3b. For each experiment, movement times for hand- and foot-related verbs are reported at each stimulus onset asynchrony.


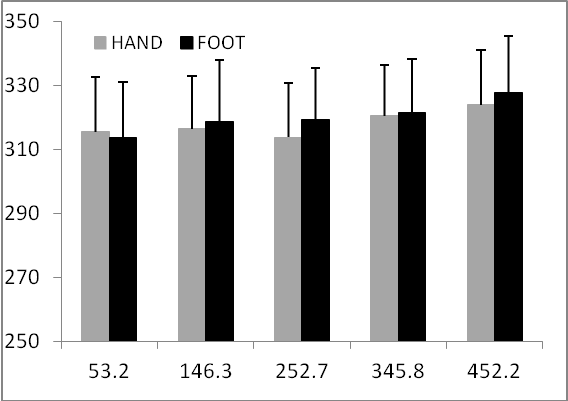


Fig 3. Effect of interference of verb category on arm movements at five different stimulus onset asynchronies (SOA) covering a time span of about 400 ms (experiment 3a). Mean movement times obtained when participants responded to hand- and foot-related verbs at each SOA are reported. Movements were executed just with the right arm.

- 1. *Experiment 3b (time course: time window of about 1000 ms)*

Results are shown in Table 2 and in fig. 4. To assess the effect of verb processing on MTs at different SOAs, we performed a 2-way repeated measures ANOVA with verb (hand, foot) and SOA (53.2, 332.5, 598.5, 864.5, 1130.5) as factors. We found a significant main effect of factor verb [F(1,12)=10.1, p<0.01] and of factor SOA [F(1.8,22.2)=13.17, p<0.001]. Movements executed when hand-related verbs were presented were slightly slower than MTs performed when foot-related verbs were presented (302.2±14.1 vs. 299.5±14.4, respectively). This result is explained by taking into account the significant interaction between verb and SOA [F(4,48)=2.68, p<0.05]. In fact, pairwise comparisons showed that hand-related verbs were significantly slower than foot-related verbs only at the shortest SOA (290.1±14.7 vs. 276.8±16.1).

In conclusion, differently from what we obtained for the RTs, in this experiment MTs the SOA at which the interference effect is present was the shortest one.


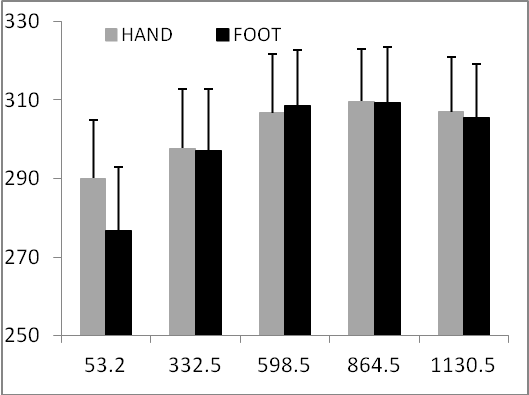


Fig 4. Effect of interference of verb category on arm movements at five different stimulus onset asynchronies (SOA) covering a time span of about 1000 ms (experiment 3b). Mean movement times obtained when participants responded to hand- and foot-related verbs at each SOA are reported. Movements were executed just with the right arm.

- 1. *Experiment 4 (color discrimination task)*

Results are shown in Table 1 and in fig. 5. To assess the effect of verb processing on MTs in this experiment, we ran a 2-way repeated measures ANOVA with verb (hand, foot, abstract) and SOA (53.2, 332.5) as factors. We found only a main effect of the factor SOA [F(1,11)=8.19, p<0.05], as MT were significantly faster when movements were made after the shortest SOA than after the longest SOA (306.5±19.2 vs.. 319.5±19.9, respectively). Neither the factor verb [F(2,22)=1.34, p=0.28] or the interaction [F(2,22)=0.26, p=0.77] were significant.


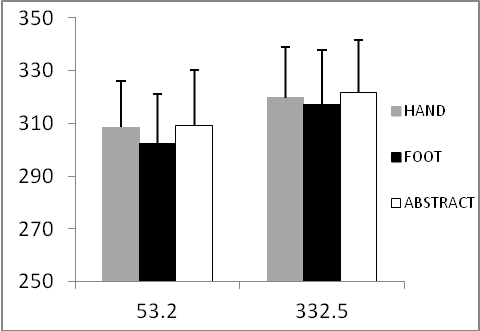


Fig 5. **Effect of interference of verb category on arm movements in the color discrimination task (experiment 4)**. Mean movement times obtained when participants responded to hand-related, foot-related and abstract verbs at the stimulus onset asynchrony (SOA) of 53.2 ms and at SOA of 332.5 ms. Movements were executed just with the right arm.

All in all, MTs have a pattern which does not overlap with that of RTs and mistakes. This might be due either to the greater variability of MTs with respect to that of the other two variables or to the fact that neural processes occurring during the execution of movements are different from those occurring during their planning. Definitely further studies are needed to clarify this issue.
